# ERICA: prevalence of asthma in Brazilian adolescents

**DOI:** 10.1590/S01518-8787.2016050006682

**Published:** 2016-02-02

**Authors:** Fábio Chigres Kuschnir, Ricardo Queiroz Gurgel, Dirceu Solé, Eduardo Costa, Mara Morelo Rocha Felix, Cecília Lacroix de Oliveira, Maurício Teixeira Leite de Vasconcellos, Maria Cristina Caetano Kuschnir

**Affiliations:** I Departamento de Pediatria. Faculdade de Ciências Médicas. Universidade do Estado do Rio de Janeiro. Rio de Janeiro, RJ, Brasil; II Departamento de Medicina. Universidade Federal de Sergipe. Aracaju, SE, Brasil; III Disciplina de Alergia Imunologia Clínica e Reumatologia. Departamento de Pediatria. Universidade Federal de São Paulo. São Paulo, SP, Brasil; IV Departamento de Medicina Interna. Faculdade de Ciências Médicas. Universidade do Estado do Rio de Janeiro. Rio de Janeiro, RJ, Brasil; V Programa de Pós-Graduação em Ciências Médicas. Faculdade de Ciências Médicas. Universidade do Estado do Rio de Janeiro. Rio de Janeiro, RJ, Brasil; VI Departamento de Nutrição Aplicada. Instituto de Nutrição. Universidade do Estado do Rio de Janeiro. Rio de Janeiro, RJ, Brasil; VII Escola Nacional de Ciências Estatísticas. Fundação Instituto Brasileiro de Geografia e Estatística. Rio de Janeiro, RJ, Brasil; VIII Núcleo de Estudos de Saúde do Adolescente. Faculdade de Ciências Médicas. Universidade do Estado do Rio de Janeiro. Rio de Janeiro, RJ, Brasil

**Keywords:** Adolescent, Asthma, epidemiology, Prevalence, Cross-Sectional Studies

## Abstract

**OBJECTIVE:**

To describe the prevalence of asthma and physician-diagnosed asthma in Brazilian adolescents.

**METHODS:**

Cross-sectional, national, school-based study with adolescents from 12 to 17 years old, participants in the Study of Cardiovascular Risks in Adolescents (ERICA). The study stratified the sample by region and grouped according to schools and classes with representativeness to the set of cities with more than 100,000 inhabitants of the Country, macro-regions, capitals, and Federal District. A questionnaire collected data through a self-filled in method. We calculated the prevalences and their confidence intervals of 95% (95%CI) according to sex, age group, type of school and skin color.

**RESULTS:**

Between 2013 and 2014, 74,589 adolescents were evaluated, 55.3% of the female sex. The total prevalence of active asthma was of 13.1% (95%CI 12.1-13.9), being higher in girls (14.8%; 95%CI 13.7-16.0) when compared to boys (11.2%; 95%CI 10.3-12.2) in all geographical strata examined. It was also higher between students of private schools (15.9%; 95%CI 14.2-17.7) when compared to public ones (12.4%; 95%CI 11.4-13.4). It was higher in the Southeast region (14.5%; 95%CI 12.9-16.1), and in the city of Sao Paulo (16.7%; 95%CI 14.7-18.7). The lowest prevalence was observed in North region (9.7%; 95%CI 9.7-10.5), and in Teresina (6.3%; 95%CI 4.9-7.7). The prevalence of physician-diagnosed asthma was of 8.7% (95%CI 8.2-9.1); higher in the North region (13.5%; 95%CI 12.7-14.2), and in Porto Alegre (19.8%; 95%CI 17.5-22.3). It was lower in the Midwest (6.9%; 95%CI 6.0-7.8), and in Cuiaba (4.8%; 95%CI 3.8-5.9). We found no significant difference in the expression of this rate between the sexes, as well as in other variables evaluated by the study.

**CONCLUSIONS:**

The prevalence of asthma in Brazilian adolescents is high. Rates of active asthma and physician-diagnosed asthma vary widely in different regions and capitals evaluated by the ERICA. These results may assist in the preparation of preventive programs and policies on health and a better understanding of the factors associated with asthma in this age group.

## INTRODUCTION

Asthma is a heterogeneous disease, generally characterized by chronic inflammation of the lower airways. The history of respiratory symptoms defines the disease, such as wheezing, shortness of breath, chest tightness, and cough. These symptoms vary over time and in intensity, along with a variable limitation of the expiratory airflow^10^. Considered the most common non-transmissible chronic disease of childhood and adolescence, the estimated number of asthmatics is approximately 300 million people worldwide, with projection to 2025 of about 400 million. Approximately 250,000 deaths attributable to asthma occur annually worldwide, and most of them are preventable^10^ ^,^ ^16^ ^,^ ^24^.

The absence of a definition of asthma with high sensitivity and specificity that could be used in epidemiologic investigations has been a challenge in studies of the disease^18^. The International Study of Asthma and Allergies in Childhood (ISAAC), conceived in the 1990’s, was a milestone in the epidemiological study of asthma and other allergic diseases. By using standardized questionnaires, it allowed valid comparisons of prevalence and severity of asthma among children and adolescents from different cities and countries^1^.

The first phase of ISAAC, finalized in 1996, involved about 450,000 adolescents of 13-14 years old from 155 different collaborating centers, located in 56 countries. The overall average prevalence of asthma, defined by the presence of wheezing in the last 12 months, was of 13.2%. On that occasion, Brazil presented the eighth greatest prevalence (19.5%) among all the centers participating in the study^12^.

ISAAC phase III was held seven years after the first phase and had as participants almost a million adolescents of 13-14 years from 233 centers in 97 countries. This phase has documented the increase in global average prevalence of asthma to 13.7%^17^. Both worldwide, and considering only Latin America, developing or developed countries, and urban areas presented the highest prevalence of the disease, while the lowest rates were in least developed countries and rural areas^6^ ^,^ ^14^.

Between the years of 2002-2003, 21 centers from the five macro-regions of Brazil participated in the ISAAC phase III, comprising 58,144 adolescents of 13-14 years old. The average prevalence of active asthma in this population was of 19.0%, varying between 11.8% and 30.5%^20^. Comparative data of adolescents from five Brazilian cities participating in phases I and III of ISAAC, published in 2006, showed discrete reduction of the prevalence of active asthma in this age group^21^.

The *Pesquisa Nacional em Saúde do Escolar* (PeNSE – National Survey on Students’ Health) occurred in 2012 and had the participation of 109,104 Brazilian adolescents from the ninth-grade of the elementary school, the majority (86.0%) aged between 13 and 15 years old. PeNSE also used the ISAAC protocol asthma module to estimate the prevalence of the disease. Global rates of 23.2% and 12.4% were found, respectively, for asthma and physician-diagnosed asthma^2^.

Asthma is a public health issue in children and adolescents, and the determination of its current dimension in the country will be able to assist in the preparation of preventive programs and health policies aimed at the disease. In 2013-2014, the Study of Cardiovascular Risks in Adolescents (ERICA) was performed, whose primary purpose was to estimate the prevalence of these risk factors in this age group^4^. Here we described the prevalence of asthma and physician-diagnosed asthma in Brazilian adolescents.

## METHODS

A descriptive study using data from ERICA, a cross-sectional, national school-based study performed in 2013 and 2014. The sample was composed of 74,589 adolescents of both sexes from 12 to 17 years old, enrolled in public and private schools of 273 cities with more than 100,000 inhabitants from all units of the Federation of Brazil^4^.

The study population was divided into 32 strata, referring to 27 capital cities, and five strata comprising the remaining cities of more than 100,000 inhabitants of each of the Brazilian macro-regions. After the geographical stratification, we performed selections by schools and classes. The schools were selected in each geographical stratum with probability proportional to their number of eligible students in the grades (seventh, eighth and ninth of elementary school and three grades of high school) and inversely proportional to the distance between the city and the capital of the federative unit. In the second stage, three combinations of shift (morning and afternoon) were selected and grade eligible in each sample school. In the third stage, one of each combination of shift and grade was selected by equiprobability. In each selected class, all students were invited to participate in the research. Adolescents with physical disabilities who made the anthropometric assessment impossible and pregnant adolescents were excluded from the study.

Thus, the sample was representative for the set of teenagers without disability and non-pregnant women studying in schools in the cities of more than 100,000 inhabitants in the national and regional level as well as for each capital. The expansion values of the sample took the non-response rate into consideration. The full description of the sample design can be found in a prior publication^25^.

The adolescents self-filled the questionnaires, which comprised sociodemographic data, questions about work, physical activity, eating behavior, smoking, consumption of alcohol, oral health, common mental disorders, reproductive health, medical records of chronic diseases, and sleep. The data were collected using microcomputers (personal digital assistant – PDA), supervised by team of previously trained professionals for field application of the standard techniques of the study. The variables on asthma were extracted from the written questionnaire and standardized asthma module of ISAAC to the age range of 13-14 years old, translated and validated for the Brazilian Portuguese^1^ ^,^ ^19^ ^,^ and measured by using the following questions:

- “In the last 12 months (one year), how many attacks of wheezing did you have?” (never had bouts of wheezing; no attacks in the last 12 months; one to three attacks; four to 12 attacks; more than 12 attacks; I don’t know or don’t remember).
- "Did a doctor tell you that you have asthma?” (Yes; no; I don’t know).

Those who reported at least one wheezing attacks in the last 12 months have been diagnosed as having active asthma. The presence of wheezing in the past 12 months shows high sensitivity and specificity (88.0% and 90.0%, respectively), compared to the evaluation of bronchial reactivity by provocation using methacholine, considered the gold standard for diagnosis of asthma, according to a validation study performed in Brazil^5^. The presence of asthma diagnosed by a physician was defined by the percentage of positive responses to the specific question.

The prevalence of asthma and physician-diagnosed asthma, and their respective confidence intervals of 95% (95%CI) were estimated for the set of cities with more than 100,000 inhabitants in Brazil, large geographical regions, and capitals, being stratified by sex, age in years (12 to 14, and 15 to 17), skin color, and type of school (public or private).

We analyzed the data in the Stata software, version 14.0, using the set of commands for analyzing complex survey data in the sample.

The study followed the principles of the Declaration of Helsinki and was approved by the Research Ethics Committees of the Institute of Studies in Public Health at the Universidade Federal do Rio de Janeiro (Process 452,008 dated November 2, 2009), and of the 26 states and the Federal District. Each participant student signed the term of consent and, additionally, the parents signed the informed consent form according to individual requirement of the Ethics Committees of the participating institutions. Student privacy and confidentiality of the data is guaranteed throughout the study.

## RESULTS

We analyzed data from 74,589 adolescents. Considering the calibrated sampling weights, 55.3% of respondents were female, 45.7% between 12 and 14 years old, and the others, between 15 and 17 years old. As to the type of school, 78.7% studied in public schools, and 21.3% in private schools (Table 1), and almost all schools surveyed (98.2%) were located in urban areas. As for skin color, 50.9% declared being of a mixed race, 35.5% as being white, and 7.6% as black. The percentage of Indigenous or Asian participants was less than 3.0%. The total percentage of participants that did not respond to the ERICA questionnaire was of 27.1%, varying from 30.0% in Midwest region to 20.0% in the South region. Eligible students who did not participate in the study were mostly of a higher age group, from public schools, and male (Table 1).

**Table 1:** Prevalence (%) of asthma and physician-diagnosed asthma in Brazilian adolescents according to macro-regions, sex, age, and type of school network. ERICA, 2013-2014.

Variable	Sample	Estimated population	Did not answer questionnaire	Active asthma		Physician-diagnosis	
		
			%	%	95%CI	%	95%CI
Macro-regions							
North	15,073	855,362	27.0	9.7	9.0-10.5	13.5	12.7-14.2
Northeast	23,167	2,165,033	27.0	10.1	8.8-11.4	9.0	8.1-9.9
Midwest	9,727	778,010	31.7	13.6	11.9-15.3	6.9	6.0-7.8
Southeast	17,080	5,153,506	28.6	14.5	12.9-16.1	7.6	6.8-8.3
South	9,542	1,195,789	19.0	13.9	12.5-15.3	10.4	8.9-11.9
Sex							
Female	41,225	5,052,137	24.2	14.8	13.7-16.0	8.5	7.8-9.1
Male	33,364	5,095,563	30.4	11.2	10.3-12.2	8.9	8.2-9.5
Age (in years)							
12-14	34,141	5,348,201	22.4	12.6	11.3-13.9	8.9	8.3-9.5
15-17	40,448	4,799,499	30.6	13.4	12.3-14.6	8.4	7.7-9.1
Type of school							
Private	15,882	1,765,446	21.6	15.9	14.2-17.7	9.6	8.7-10.5
Public	58,707	8,382,253	28.3	12.4	11.4-13.4	8.5	7.9-9.0
Brazil	74,589	10,147,700	27.1	13.1	12.1-13.9	8.7	8.2-9.1

The overall prevalence of active asthma was of 13.1% (95%CI 12.1-13.9), being higher in the female sex (14.8%; 95%CI 13.7-16.0) when compared to the male sex (11.2%; 95%CI 10.3-12.2) and in private network students (15.9%; 95%CI 14.2-17.7) when compared to the public network students (12.4%; 95%CI 11.4-13.4). These rates were similar in both studied age groups. The overall prevalence of asthma medical diagnosis was of 8.7% (95%CI 8.2-9.1), without significant differences between sex, age, and skin color. As well as active asthma, its prevalence was highest among adolescents of the private network, but without achieving statistical significance in relation to public schools students (Table 1).

The prevalence of active asthma was higher in the Southeast region (14.5%; 95%CI 12.9-16.1) and in the cities of Sao Paulo (16.7%; 95%CI 14.7-18.7); Belo Horizonte (15.8%; 95%CI 13.9-17.7), and Goiania (15.4%; 95%CI 13.1-17,7). Among the macro-regions, the North region exhibited the lowest prevalence (9.7%; 95%CI 9.7-10.5), as well as the cities of Teresina (6.3%; 95%CI 4.9-7.7); Sao Luis (7.4%; 95%CI 6.0-8.8), and Joao Pessoa (7.8%; 95%CI 6.4-9.2). The prevalence of active asthma was higher in females in all the capitals and regions of Brazil (Tables 1 and 2).

**Table 2:** Prevalence (%) of active asthma in adolescents from 12 to 17 years old by sex, according to the Brazilian state capitals. ERICA, 2013-2014.

States capital	Sample	Estimated population	Active asthma		Female		Male	
		
			%	95%CI	%	95%CI	%	95%CI
Porto Velho	1,349	5,2526	11.0	8.1-13.9	12.9	9.5-16.3	9.1	5.3-12.8
Rio Branco	1,740	39,603	8.9	7.4-10.4	12.3	10.1-14.5	5.4	4.0-6.9
Manaus	3,549	214,042	10.2	9.1-11.3	12.4	10.6-14.2	8.0	6.7-9.3
Boa Vista	750	35,813	11.5	8.4-14.7	12.7	8.1-17.2	10.4	5.0-15.8
Belem	2,329	138,765	10.6	9.0-12.2	12.0	10.3-13.8	9.1	7.1-11.1
Macapa	1,370	52,573	12.9	10.7-15.1	16.4	13.0-19.9	9.2	6.6-11.8
Palmas	1,170	27,928	10.9	8.3-13.6	12.6	9.0-16.2	9.3	6.6-11.9
Sao Luis	2,577	105,548	7.4	6.0-8.8	8.6	7.0-10.1	6.2	4.2-8.2
Teresina	1,733	83,365	6.3	4.9-7.7	8.0	5.8-10.3	4.6	2.9-6.4
Fortaleza	2,665	244,925	10.3	9.0-11.6	12.1	10.3-13.9	8.5	6.5-10.4
Natal	1,944	79,819	10.9	8.5-13.2	13.6	11.2-16.0	8.2	5.4-10.9
Joao Pessoa	1,956	70,050	7.8	6.4-9.2	9.0	7.2-10.80	6.7	4.9-8.4
Recife	2,534	896,251	9.6	7.3-11.9	9.8	9.3-10.2	3.5	3.1-3.9
Maceio	2,082	101,491	8.0	6.4-9.7	9.7	7.3-12.2	6.4	4.4-8.4
Aracaju	1,788	59,576	9.4	7.2-11.7	10.3	6.8-13.9	8.6	6.3-10.8
Salvador	1,890	252,397	8.4	6.4-10.5	10.2	7.6-12.8	6.6	3.9-9.4
Campo Grande	1,223	78,769	11.0	8.6-13.4	11.6	8.4-14.9	10.3	6.4-14.2
Cuiaba	1,910	52,153	11.5	9.8-13.2	13.6	11.3-15.9	9.6	6.9-12.2
Goiania	1,598	125,057	15.4	13.1-17.7	16.4	13.9-18.9	14.5	10.5-18.4
Brasilia	2,689	268,118	14.8	12.6-17.1	16.2	14.2-18.3	13.4	9.8-17.0
Belo Horizonte	2,569	203,990	15.8	13.9-17.7	19.0	6.7-21.3	12.6	10.5-14.8
Vitoria	1,372	29,237	11.7	9.8-13.5	12.4	10.0-14.9	10.9	8.2-13.5
Rio de Janeiro	3,516	516,063	8.9	7.7-10.0	10.7	8.6-12.7	7.1	5.2-9.0
Sao Paulo	3,700	980,486	16.7	14.7-18.7	19.8	16.3-23.3	13.6	12.0-15.2
Curitiba	2,532	156,140	14.6	13.1-16.2	15.8	13.5-18.0	13.5	10.9-16.1
Florianopolis	1,145	36,420	12.5	9.0-16.0	15.0	10.8-19.2	10.0	6.7-13.2
Porto Alegre	1,948	114,959	14.9	12.9-16.8	17.6	14.4-20.9	12.2	9.9-14.4

**Figure 1:**
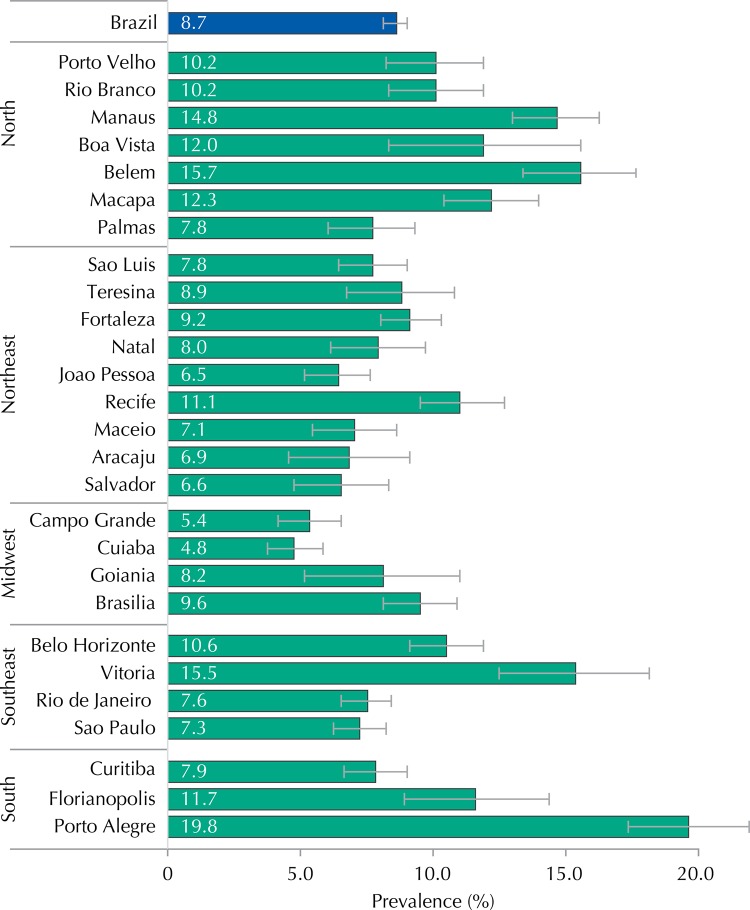
Prevalence (%) and 95%CI of physician-diagnosed asthma in adolescents of 12-17 years old according to the Brazilian state capitals. ERICA, 2013-2014.

Regarding the physician-diagnosed asthma, the highest prevalence was observed in the North region (13.5%; 95%CI 12.7-14.2), and in the cities of Porto Alegre (19.8%; 95%CI 17.5-22.3), Belem (15.7%; 95%CI 13.5-17.8), and Vitoria (15.5%; 95%CI 12.6-18.3). On the other hand, the Midwest region exhibited the lowest prevalence among the macro-regions (6.9%; 95%CI 6.0-7.8), as well as the cities of Cuiaba (4.8%; 95%CI 3.8-5.9); Campo Grande (5.4%; 95%CI 4.2-6.6), and Joao Pessoa (6.5%; 95%CI 5.2-7.7) (Table 1 and Figure 1).

The distribution of the prevalence of physician-diagnosed asthma per sex varied widely between different geographical strata studied. These rates were higher in males in the North (14.6% *versus* 12.9%), Northeast (9.9% *versus* 8.2%), Midwest (7.9% *versus* 5.9%), and South (10.7% *versus* 10.1%) regions, while in the Southeast the prevalence was of females (7.8% *versus* 7.3%), without achieving statistical significance. However, in the total sample, the physician-diagnosed asthma was significantly higher in females (14.8%; 95%CI 13.7-16.0) when compared to the males (11.2%; 95%CI 10.3-12.2).

Regarding skin color, the prevalence of active asthma was higher in those of white color (14.9%; 95%CI 12.9-16.8) when compared to black color (10.8%; 95%CI 9.2-12.3), and mixed-race people (12.1%; 95%CI 11.2-12.9), without any differences in the distribution of the prevalence of physician-diagnosed asthma.

## DISCUSSION

The results of ERICA showed an average prevalence of active asthma of 13.1% in adolescents from 12 to 17 years old, ranging from 6.3% in Teresina and 16.7% in Campo Grande, and with predominance among females in every geographic strata. Regarding the regions, the variation was of between 9.7% in the North region, and 14.5% in the Southeast region.

The average prevalence of physician-diagnosed asthma in the cities of more than 100,000 inhabitants in Brazil was of 8.7%, with a variation of 6.9% in the Midwest region up to 13.5% in the Northern region. This difference was even more pronounced among the capitals, 4.8% in Cuiaba to 19.8% in Porto Alegre, but without any difference between the sexes. The study considered the percentage of non-responses to the questionnaire study satisfactory and its variability may have been due to the requirement of informed consent form for some states, with observed percentages as the lowest participation.

The comparative analysis between the data obtained and the one previously observed by the ISAAC phase III study in some locations in Brazil showed a reduction in prevalence rates of asthma and physician-diagnosed asthma^20^. In a later study (in 2012), Solé et al., using the same method, have checked the prevalence of asthma in adolescents of 13-14 years old in seven cities participating in the ISAAC phase III in 2003 and its temporal trend after nine years of study. During this period, a drop of average active asthma prevalence was observed (19.5% *versus* 17.5%) with an increase of physician-diagnosed asthma (14.3% *versus* 17.6%), both higher than those obtained by ERICA^23^.

In PeNSE 2012, the rates of 23.2% and 12.4% were respectively for active asthma and physician-diagnosed asthma. Similar to our findings, the largest percentages of active asthma (24.9% *versus* 14.5%) and physician-diagnosed asthma (18.4% *versus* 13.5%) were observed respectively between students from the Southeast and Northern regions. On the other hand, unlike ERICA, the lowest percentages for these prevalences in PenSE 2012 occurred respectively in the Northeast (19.8%) and Southeast regions (11.4%)^2^.

**Figure 2:**
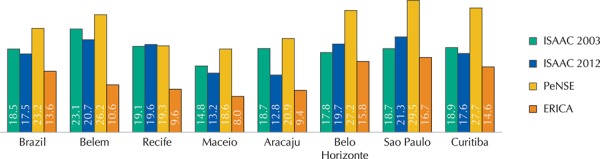
Prevalence (%) of active asthma in adolescents who participated in the ISAAC phase III (2003/2012), PeNSE, and ERICA.

ISSAC: International Study of Asthma and Allergies in Childhood; PeNSE: National Survey on Students’ Health; ERICA: Study of Cardiovascular Risks in Adolescents

**Figure 3:**
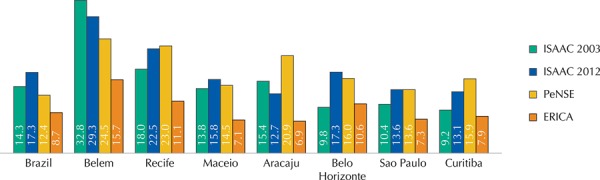
Prevalence (%) of physician-diagnosed asthma in adolescents who participate in the ISAAC phase III (2003/2012), PeNSE, and ERICA.

ISSAC: International Study of Asthma and Allergies in Childhood; PeNSE: National Survey on Students’ Health; ERICA: Study of Cardiovascular Risks in Adolescents

The prevalence of asthma and physician-diagnosed asthma in Brazil, in seven Brazilian cities evaluated by ERICA, and participants of the ISAAC phase III and reevaluated in 2012, and included in the PeNSE are shown in Figures 2 and 3.

A superficial analysis would allow for the conclusion about the time reduction of these rates after previous significant elevation (Figure 2). However, some factors should be considered in the analysis. First, the age group evaluated by ERICA is wider than that of ISAAC and PeNSE, and the input of older adolescents could generate dilution in the rates observed. However, we did not find no significant differences between the rates of prevalence of asthma among the active participants of ERICA older than 15 years old and those between 12 and 14 years old, closer to the populations of the other studies cited.

Another aspect relates to the way the question about asthma in our study was rebuilt, different than the one presented in ISAAC’s asthma module^1^. The original question about the presence of “wheezing in the past 12 months”, useful for the assessment of asthma prevalence^1^ differed from our question where we asked about the number of “wheezing attacks in the past 12 months”. The frequency of attacks in the last year, present as an independent question in ISAAC’s asthma module, is considered useful for the assessment of the severity of the disease, offering an alternative quantitative measure of frequency of wheezing^1^. Thus, we include the term “attacks” for the epidemiological diagnosis of active asthma. The sensitivity to this issue may have been reduced in relation to the original one, which can make it difficult to compare our results with those from other studies. On the other hand, those who responded positively to this question may represent a portion of adolescents with clinical picture of greater severity or worse disease control, requiring greater care and exerting greater burden on the health system.

The difficulty in diagnosing milder clinical pictures of asthma in adolescents can cause uncertainty about the real extent of the disease in this age group^3^. Longitudinal studies have shown that a significant percentage of individuals, generally those with more severe asthma in childhood, remain symptomatic during adolescence and will be asthmatic adults^11^. Currently, different asthma phenotypes have been recognized including in the beginning of adolescence, in defiance of common sense that asthma has atopic origin and improves during puberty^7^. Besides, when we work with adolescents we should pay attention to some aspects not specifically linked to asthma, as the degree of perception of health and disease, as well as the cognitive differences between the sexes can influence the results of studies in this population.

The prevalence of physician-diagnosed asthma obtained by ERICA was also lower when compared to the results of ISAAC and PeNSE (Figure 3). However, the relationship between the higher prevalence of asthma and the physician-diagnosed asthma keeps its proportion in the four studies, which agrees with other national and international studies^3^ ^,^ ^15^.

The positive response to the question “Have you ever had asthma in your life?”, original from the ISAAC protocol asthma module, is considered as a possible diagnosis by a physician for this condition^1^. The difference between this rate and the active asthma rate has been interpreted as a medical misdiagnosis of asthma, which could result in the delay or the lack of treatment for the disease^3^. Another aspect that may affect the interpretation of this issue is the variation in the terminology and the concepts of the same disease to different populations and health professionals, often influenced by cultural factors^a^. In ERICA we asked the following question: “Did a doctor tell you that you have asthma?”, a more objective question that might justify its lower prevalence in comparison with other studies presented in Figure 3. The percentage of positive responses to this question in our study may translate more accurately the real dimension to the access to medical services, and justify the great variation found to this rate among the capitals studied.

Similarly to other studies^9^ ^,^ ^13^ without the amplitude of ERICA, we observed higher prevalence of active asthma among female adolescents. In general, boys have a higher chance of developing wheezing and asthma in the early years of life. However, this risk reduces gradually until the end of childhood, a time in which the disease becomes more prevalent among girls. Although recognized, the association between female sex and asthma in adolescence does not have a well-established cause to date. The study has mentioned possible factors like the size of the airways, hormonal influences, and overweight in preschool years, psychosocial aspects, and environmental exposures specific to each sex. In addition, epidemiological studies with adolescents have shown possible differences in the level of interest for filling in questionnaires, with boys tend to underestimate and the girls tend to overestimate their symptoms^9^.

In contrast with the results of the PeNSE^2^, which showed no significant differences between public and private school students, we found a higher percentage of active asthma among adolescents from private schools. In ERICA, where the majority of the sample is from public schools, the enrollment in private school can mean better financial condition of the family and greater access to health services, or even a better perception of the disease with greater interest to participate in the study. Although no significant association between socioeconomic status and the prevalence of the disease in Brazilian cities participating in the ISAAC III have been found^22^, our results may reflect social inequalities in this population.

In a Country like Brazil, of continental dimensions, regional, cultural and socioeconomic differences can coexist with great variation of environmental factors. A study conducted in seven cities of the Northeast region participating in the ISAAC phase III assessed the association between the presence of active asthma and different socioeconomic indicators and environmental conditions. We observed a positive association between the prevalence of asthma and water deprivation and the latitude from the center, and inverse association with the annual average temperature^8^. Similarly, we observed increased prevalence of asthma in the South of the Country.

The results of ERICA show that the prevalence of asthma remains very high in Brazilian adolescents, constituting an important public health problem in this age group. The prevalence rates of asthma and medical diagnosis of the disease vary widely in different regions and capitals evaluated by the study. The future investigation on the regional factors associated with asthma may improve the understanding of its natural history, its demographic determinants, and the differences in access to medical care. They can also collaborate to the development of preventive programs and health policies geared to the disease in adolescents.

## Footnotes

a Aires ST. Emprego dos termos asma e bronquite em inquérito epidemiológico sobre doença sibilante entre escolares [dissertation]. Rio de Janeiro (RJ): Faculdade de Medicina da Universidade Federal do Rio de Janeiro; 2005.

## References

[1] Asher MI, Keil U, Anderson HR, Beasley R, Crane J, Martinez FD (1995). International Study of Asthma and Allergies in Childhood (ISAAC): rationale and methods. Eur Respir J.

[2] Barreto ML, Ribeiro-Silva RC, Malta DC, Oliveira-Campos M, Andreazzi MA, Cruz AA (2014). Prevalência de sintomas de asma entre escolares do Brasil: Pesquisa Nacional em Saúde do Escolar (PeNSE 2012). Rev Bras Epidemiol.

[3] Bisgaard H, Szefler SJ (2005). Understanding mild persistent asthma in children: the next frontier. J Allergy Clin Immunol.

[4] Bloch KV, Szklo M, Kuschnir MC, Abreu GA, Barufaldi LA, Klein CH (2015). The Study of Cardiovascular Risk in Adolescents – ERICA: rationale, design and sample characteristics of a national survey examining cardiovascular risk factor profile in Brazilian adolescents. BMC Public Health.

[5] Camelo-Nunes IC, Wandalsen GF, Melo KC, Naspitz CK, Solé D (2001). Prevalência de asma e de sintomas relacionados entre escolares de São Paulo, Brasil: 1996 a 1999: estudo da reatividade brônquica entre adolescentes asmáticos e não asmáticos – International Study of Asthma and Allergies in Childhood (ISAAC). Rev Bras Alergia Imunopatol.

[6] Cooper PJ, Rodrigues LC, Cruz AA, Barreto ML (2009). Asthma in Latin America: a public health challenge and research opportunity. Allergy.

[7] Ford ES (2005). The epidemiology of obesity and asthma. J Allergy Clin Immunol.

[8] Franco JM, Gurgel R, Solé D, França VL, Brabin B, Brazilian ISAAC Group (2009). Socio-environmental conditions and geographical variability of asthma prevalence in Northeast Brazil. Allergo lmmunopathol (Madr).

[9] Fuhlbrigge AL, Jackson B, Wright RJ (2002). Gender and asthma. Immunol Allergy Clin N Am.

[10] Global Initiative for Asthma (GINA) (2015). Global Strategy for Asthma Management and Prevention: updated 2015.

[11] Guerra S, Wright AL, Morgan WJ, Sherrill DL, Holberg CJ, Martinez FD (2004). Persistence of asthma symptoms during adolescence: role of obesity and age at the onset of puberty. Am J Respir Crit Care Med.

[12] International Study of Asthma and Allergies in Childhood (ISAAC) Steering Committee (1998). Worldwide variation in prevalence of symptoms of asthma, allergic rhinoconjunctivitis, and atopic eczema: ISAAC. Lancet.

[13] Kuschnir FC, Alves da Cunha AJ (2007). Environmental and socio-demographic factors associated to asthma in adolescents in Rio de Janeiro, Brazil. Pediatr Allergy Immunol.

[14] Lai CK, Beasley R, Crane J, Foliaki S, Shah J, Weiland S (2009). Global variation in the prevalence and severity of asthma symptoms: phase three of the International Study of Asthma and Allergies in Childhood (ISAAC). Thorax.

[15] Mallol J, Solé D, Asher I, Clayton T, Stein R, Soto-Quiroz M (2000). Prevalence of asthma symptoms in Latin America: The International Study of Asthma and Allergies in Childhood (ISAAC). Pediatr Pulmonol.

[16] Pawankar R, Baena-Cagnani CE, Bousquet J, Canonica GW, Cruz AA, Kaliner MA (2008). State of world allergy report 2008: allergy and chronic respiratory diseases. World Allergy Organ J.

[17] Pearce N, Aït-Khaled N, Beasley R, Mallol J, Keil U, Mitchell E (2007). Worldwide trends in the prevalence of asthma symptoms: phase III of the International Study of Asthma and Allergies in Childhood (ISAAC). Thorax.

[18] Peat JK, Toelle BG, Marks GB, Mellis CM (2001). Continuing the debate about measuring asthma in populations studies. Thorax.

[19] Solé D, Vanna AT, Yamada E, Rizzo MC, Naspitz CK (1998). International Study of Asthma and Allergies in Childhood (ISAAC) written questionnaire: validation of the asthma component among Brazilian children. J Invest Allergol Clin Immunol.

[20] Solé D, Wandalsen GF, Camelo-Nunes IC, Naspitz CK, ISAAC - Brazilian Group (2006). Prevalence of asthma; rhinitis; and atopic eczema among Brazilian children and adolescents identified by the International Study of Asthma and Allergies in Childhood (ISAAC) - Phase 3. J Pediatr (Rio J).

[21] Solé D, Melo KC, Camelo-Nunes IC, Freitas LS, Britto M, Rosário NA (2007). Changes in the prevalence of asthma and allergic diseases among Brazilian schoolchildren (13-14 years old): comparison between ISAAC Phases One and Three. J Trop Pediatr.

[22] Solé D, Camelo-Nunes IC, Wandalsen GF, Mallozi MC, Naspitz CK (2008). Is the prevalence of asthma and related symptoms among Brazilian children related to socioeconomic status?. J Asthma.

[23] Solé D, Rosário NA, Sarinho ES, Camelo-Nunes IC, Barreto BAP, Medeiros ML (2015). Prevalência de asma e doenças alérgicas em adolescentes: estudo evolutivo de nove anos (2003 a 2012). J Pediatr (Rio J).

[24] The Asthma Network (GAN) (2014). The Global Asthma Report 2014.

[25] Vasconcellos MTL, Silva PLN, Szklo M, Kuschnir MCC, Klein CH, Abreu GA (2015). Desenho da amostra do Estudo de Risco Cardiovascular em Adolescentes (ERICA). Cad Saude Publica.

