# ERRATA

**DOI:** 10.1590/S01518-8787.2016050006682err

**Published:** 2016-04-15

No artigo “**ERICA: prevalência de asma em adolescentes brasileiros**”

DOI: 10.1590/S01518-8787.2016050006682, publicado no periódico “Revista de Saúde Pública”, volume 50 de 2016, suplemento ERICA, artigo 13s, na página 3, nos items “a” e “b”.

**Onde se lê:**

a) “In the last 12 months (one year), how many attacks of wheezing did you have?” (never had bouts of wheezing; no attacks in the last 12 months; one to three attacks; four to 12 attacks; more than 12 attacks; I don’t know or don’t remember).

b) “Did a doctor tell you that you have asthma?” (Yes; no; I don’t know).

**Leia-se:**

a) “Nos últimos 12 meses (um ano), quantas crises de sibilos (chiado no peito) você teve?” (nunca tive crises de sibilos (chiado no peito); nenhuma crise nos últimos 12 meses; uma a três crises; quatro a 12 crises; mais de 12 crises; não sei ou não lembro).

b) “Algum médico lhe disse que você tem asma?” (sim; não; não sei).

