# Supplementary material for: ERICA: prevalence of asthma in Brazilian adolescents
Source: Rev Saude Publica. 2016 Feb 2;50(Suppl 1):13s. doi: 10.1590/S01518-8787.2016050006682 (PMC4767043; doi:10.1590/S01518-8787.2016050006682)
Supplement: Translation

# Translation: ERICA: prevalência de asma em adolescentes brasileiros

**Authors:** Fábio Chigres Kuschnir, Ricardo Queiroz Gurgel, Dirceu Solé, Eduardo Costa, Mara Morelo Rocha Felix, Cecília Lacroix de Oliveira, Maurício Teixeira Leite de Vasconcellos, Maria Cristina Caetano Kuschnir

**Affiliations:** I Departamento de Pediatria. Faculdade de Ciências Médicas. Universidade do Estado do Rio de Janeiro. Rio de Janeiro, RJ, Brasil; II Departamento de Medicina. Universidade Federal de Sergipe. Aracaju, SE, Brasil; III Disciplina de Alergia Imunologia Clínica e Reumatologia. Departamento de Pediatria. Universidade Federal de São Paulo. São Paulo, SP, Brasil; IV Departamento de Medicina Interna. Faculdade de Ciências Médicas. Universidade do Estado do Rio de Janeiro. Rio de Janeiro, RJ, Brasil; V Programa de Pós-Graduação em Ciências Médicas. Faculdade de Ciências Médicas. Universidade do Estado do Rio de Janeiro. Rio de Janeiro, RJ, Brasil; VI Departamento de Nutrição Aplicada. Instituto de Nutrição. Universidade do Estado do Rio de Janeiro. Rio de Janeiro, RJ, Brasil; VII Escola Nacional de Ciências Estatísticas. Fundação Instituto Brasileiro de Geografia e Estatística. Rio de Janeiro, RJ, Brasil; VIII Núcleo de Estudos de Saúde do Adolescente. Faculdade de Ciências Médicas. Universidade do Estado do Rio de Janeiro. Rio de Janeiro, RJ, Brasil

**Keywords:** Adolescente, Asma, epidemiologia, Prevalência, Estudos Transversais

## Abstract

**OBJETIVO:**

Descrever a prevalência de asma ativa e de diagnóstico médico de asma em adolescentes brasileiros.

**MÉTODOS:**

Estudo transversal, nacional, de base escolar com adolescentes de 12 a 17 anos, participantes do Estudo de Riscos Cardiovasculares em adolescentes (ERICA). A amostra foi estratificada por região e conglomerada por escolas e turmas com representatividade para o conjunto de municípios com mais de 100 mil habitantes do País, macrorregiões, capitais e Distrito Federal. Os dados foram coletados em questionário autopreenchível. Foram calculadas as prevalências e respectivos intervalos de confiança de 95% (IC95%) segundo sexo, faixa etária, tipo de escola e cor da pele.

**RESULTADOS:**

Entre 2013 e 2014 foram avaliados 74.589 adolescentes, 55,3% do sexo feminino. A prevalência total de asma ativa foi 13,1% (IC95% 12,1-13,9), sendo superior nas meninas (14,8%; IC95% 13,7-16,0) em relação aos meninos (11,2%; IC95% 10,3-12,2) em todos estratos geográficos analisados, e também entre os alunos da rede privada (15,9%; IC95% 14,2-17,7) em relação aos de escolas públicas (12,4%; IC95% 11,4-13,4). Foi mais elevada na região Sudeste (14,5%; IC95% 12,9-16,1) e na cidade de São Paulo (16,7%; IC95% 14,7-18,7). A menor prevalência foi observada na região Norte (9,7%; IC95% 9,7-10,5) e em Teresina (6,3%; IC95% 4,9-7,7). A prevalência de diagnóstico médico de asma foi 8,7% (IC95% 8,2-9,1); mais elevada na região Norte (13,5%; IC95% 12,7-14,2) e em Porto Alegre (19,8%; IC95% 17,5-22,3) e mais baixa no Centro-Oeste (6,9%; IC95% 6,0-7,8) e em Cuiabá (4,8%; IC95% 3,8-5,9). Não ocorreu diferença significativa na expressão desta taxa entre os sexos, assim como nas outras variáveis avaliadas pelo estudo.

**CONCLUSÕES:**

A prevalência de asma em adolescentes brasileiros é alta. As taxas de asma ativa e de diagnóstico médico variam amplamente nas diferentes regiões e capitais avaliadas pelo ERICA. Estes resultados poderão auxiliar na elaboração de programas preventivos e de políticas de saúde e no melhor entendimento sobre os fatores associados à asma nessa faixa etária.

## INTRODUÇÃO

A asma é uma doença heterogênea, geralmente caracterizada pela inflamação crônica das vias aéreas inferiores. É definida pela história de sintomas respiratórios, como chiado, falta de ar, aperto no peito e tosse, que variam ao longo do tempo e em intensidade, juntamente com limitação variável do fluxo aéreo expiratório^10^ ^.^ Considerada a doença crônica não transmissível mais comum da infância e adolescência, estima-se que existam aproximadamente 300 milhões de indivíduos asmáticos em todo o mundo, com projeção para 2025 de cerca de 400 milhões. Aproximadamente 250.000 mortes atribuíveis à asma, a maioria preveníveis, ocorrem anualmente em nível mundial^10^ ^,^ ^16^ ^,^ ^24^ ^.^

A ausência de uma definição de asma com alta sensibilidade e especificidade que pudesse ser utilizada nas investigações epidemiológicas constituiu um desafio no estudo da doença^18^ ^.^ O *International Study of Asthma and Allergies in Childhood* (ISAAC), idealizado na década de 1990, foi um marco no estudo epidemiológico da asma e de outras doenças alérgicas. Permitiu, pelo uso de questionários padronizados, comparações válidas de prevalência e gravidade da asma entre crianças e adolescentes de diferentes cidades e países^1^.

A primeira fase do ISAAC, finalizada em 1996, envolveu cerca de 450 mil adolescentes de 13-14 anos de 155 diferentes centros colaboradores, localizados em 56 países. A prevalência média global de asma ativa, definida pela presença de sibilos (chiado no peito) nos últimos 12 meses, foi 13,2%. Nessa ocasião, o Brasil apresentou a oitava maior prevalência (19,5%) entre todos os centros participantes do estudo^12^.

O ISAAC fase III foi realizado sete anos após a primeira fase e teve como participantes quase um milhão de adolescentes de 13-14 anos oriundos de 233 centros de 97 países. Essa fase documentou o aumento na prevalência mundial média de asma ativa para 13,7%^17^. Tanto globalmente como considerando apenas a América Latina, países desenvolvidos ou em desenvolvimento e as áreas urbanas apresentaram as maiores prevalências da doença, enquanto as menores prevalências encontravam-se em países menos desenvolvidos e em áreas rurais^6^ ^,^ ^14^.

Entre os anos de 2002-2003, 21 centros das cinco macrorregiões brasileiras participaram do ISAAC fase III, compreendendo 58.144 adolescentes de 13-14 anos. A prevalência média de asma ativa nessa população foi 19,0%, variando entre 11,8% e 30,5%^20^. Dados comparativos de adolescentes de cinco cidades brasileiras participantes das fases I e III do ISAAC, publicados em 2006, mostraram discreta redução da prevalência de asma ativa nessa faixa etária^21^.

A Pesquisa Nacional em Saúde do Escolar (PeNSE) foi realizada em 2012 e contou com a participação de 109.104 adolescentes brasileiros do nono ano do ensino fundamental, a maioria (86,0%) na faixa etária de 13 a 15 anos. A PeNSE também utilizou o módulo de asma do protocolo ISAAC para estimar a prevalência da doença. Foram encontradas taxas globais de 23,2% e 12,4%, respectivamente, para asma ativa e diagnóstico médico de asma^2^.

A asma constitui problema de saúde pública em crianças e adolescentes e a determinação de sua atual dimensão no País poderá auxiliar na elaboração de programas preventivos e de políticas de saúde voltadas para a doença. Nos anos de 2013-2014, foi realizado o Estudo de Riscos Cardiovasculares em Adolescentes (ERICA), cujo objetivo principal estimar a prevalência desses fatores de risco nessa faixa etária^4^. Aqui, descrevemos a prevalência de asma ativa e de diagnóstico médico de asma em adolescentes brasileiros.

## MÉTODOS

Estudo descritivo utilizando os dados do ERICA, estudo transversal, nacional de base escolar, realizado em 2013 e 2014. A amostra foi composta por 74.589 adolescentes, de ambos os sexos, de 12 a 17 anos, matriculados em escolas públicas e privadas de 273 municípios com mais de 100 mil habitantes de todas as unidades da Federação do Brasil^4^.

A população do estudo foi dividida em 32 estratos, referentes às 27 capitais e cinco estratos compreendendo os demais municípios de mais de 100 mil habitantes de cada uma das macrorregiões brasileiras. Após a estratificação geográfica, foram realizadas seleções por escolas e turmas. As escolas foram selecionadas em cada estrato geográfico, com probabilidade diretamente proporcional ao seu número de alunos nos anos elegíveis (sétimo, oitavo e nono do ensino fundamental e os três anos do ensino médio) e inversamente proporcional à distância entre o município e a capital da unidade federativa. No segundo estágio, foram selecionadas três combinações de turno (manhã e tarde) e ano elegível em cada escola da amostra e, no terceiro estágio, foi selecionada com equiprobabilidade uma de cada combinação de turno e ano. Em cada turma selecionada, todos os estudantes foram convidados a participar da pesquisa. Foram excluídos os adolescentes com deficiências físicas que impossibilitassem a avaliação antropométrica e as adolescentes grávidas.

Desse modo, a amostra foi representativa para o conjunto de adolescentes sem incapacidade e não grávidas que estudam em escolas dos municípios de mais de 100 mil habitantes no nível nacional e regional e para cada capital. Os valores de expansão da amostra levaram em conta a taxa de não resposta. Descrição completa do desenho amostral encontra-se em publicação prévia^25^.

Os adolescentes responderam questionários autopreenchíveis, que compreenderam dados sociodemográficos, perguntas sobre trabalho, atividade física, comportamento alimentar, tabagismo, consumo de bebidas alcoólicas, saúde oral, transtornos mentais comuns, saúde reprodutiva, histórico médico de doenças crônicas e sono. A coleta de dados foi realizada empregando-se microcomputadores (*personal digital assistant* – PDA), supervisionada por equipe de campo previamente treinada para aplicação de técnicas padronizadas do estudo. As variáveis sobre asma foram extraídas do questionário escrito e padronizado do módulo de asma do ISAAC para a faixa etária de 13-14 anos, traduzido e validado para o português brasileiro^1^ ^,^ ^19^, e aferidas por meio das seguintes perguntas:

- “In the last 12 months (one year), how many attacks of wheezing did you have?” (never had bouts of wheezing; no attacks in the last 12 months; one to three attacks; four to 12 attacks; more than 12 attacks; I don’t know or don’t remember).
- “Did a doctor tell you that you have asthma?” (Yes; no; I don’t know).

Aqueles que reportaram pelo menos uma crise de sibilos (chiado no peito) nos últimos 12 meses foram diagnosticados como tendo asma ativa. A presença de sibilos nos últimos 12 meses apresenta sensibilidade e especificidade elevadas (88,0% e 90,0%, respectivamente), comparadas à avaliação da reatividade brônquica pela provocação pela metacolina, considerada padrão-ouro para diagnóstico de asma, conforme estudo de validação realizado no Brasil^5^. A presença de asma diagnosticada pelo médico foi definida pelo percentual de respostas positivas à pergunta específica.

A prevalência de asma ativa e de asma diagnosticada pelo médico e seus respectivos intervalos de confiança de 95% (IC95%) foram estimados para o conjunto de municípios com mais de 100 mil habitantes do Brasil, grandes regiões geográficas e capitais, e estratificados por sexo, faixa etária em anos (12 a 14 e 15 a 17), cor da pele e tipo de escola (pública ou privada).

As análises foram realizadas no *software* Stata, versão 14.0, utilizando o conjunto de comandos para análise de dados de inquéritos com amostra complexa (*survey*).

O estudo seguiu os princípios da Declaração de Helsinki e foi aprovado pelos Comitês de Ética em Pesquisa do Instituto de Estudos em Saúde Coletiva da Universidade Federal do Rio de Janeiro (Processo 45/2008 de 11/2/2009), e dos 26 estados e do Distrito Federal. O termo de assentimento foi obtido de cada estudante participante e, adicionalmente, o termo de consentimento livre e esclarecido assinado pelos pais foi providenciado conforme exigência individual dos Comitês de Ética das instituições participantes. A privacidade do aluno e a confidencialidade dos dados foram garantidas durante todo o estudo.

## RESULTADOS

Foram analisados os dados de 74.589 adolescentes. Considerando-se os pesos amostrais calibrados, 55,3% dos entrevistados eram do sexo feminino, 45,7% tinham entre 12 e 14 anos de idade e os demais, entre 15 e 17 anos. Quanto ao tipo de rede escolar, 78,7% estudavam em escolas públicas e 21,3% em escolas privadas (Tabela 1), e quase todas as escolas pesquisadas (98,2%) estavam localizadas em área urbana. Quanto à cor da pele, 50,9% declararam-se de cor parda, 35,5% branca e 7,6%, preta. O percentual de participantes indígenas ou de cor da pele amarela foi inferior a 3,0%. O percentual total de não resposta ao questionário ERICA foi 27,1%, variando de cerca de 30,0% na região Centro-Oeste a 20,0% na região Sul. Os alunos elegíveis que não participaram do estudo eram em sua maioria de faixa etária mais elevada, de escolas públicas e do sexo masculino (Tabela 1).

**Tabela 1:** Prevalência (%) de asma ativa e de asma diagnosticada pelo médico em adolescentes brasileiros segundo macrorregiões, sexo, faixa etária e tipo de rede escolar. ERICA, 2013-2014.

Variável	Amostra	População estimada	Não resposta ao questionário	Asma ativa		Diagnóstico médico	
		
			%	%	IC95%	%	IC95%
Macrorregiões							
Norte	15.073	855.362	27,0	9,7	9,0-10,5	13,5	12,7-14,2
Nordeste	23.167	2.165.033	27,0	10,1	8,8-11,4	9,0	8,1-9,9
Centro-Oeste	9.727	778.010	31,7	13,6	11,9-15,3	6,9	6,0-7,8
Sudeste	17.080	5.153.506	28,6	14,5	12,9-16,1	7,6	6,8-8,3
Sul	9.542	1.195.789	19,0	13,9	12,5-15,3	10,4	8,9-11,9
Sexo							
Feminino	41.225	5.052.137	24,2	14,8	13,7-16,0	8,5	7,8-9,1
Masculino	33.364	5.095.563	30,4	11,2	10,3-12,2	8,9	8,2-9,5
Faixa etária (em anos)							
12-14	34.141	5.348.201	22,4	12,6	11,3-13,9	8,9	8,3-9,5
15-17	40.448	4.799.499	30,6	13,4	12,3-14,6	8,4	7,7-9,1
Tipo de escola							
Privada	15.882	1.765.446	21,6	15,9	14,2-17,7	9,6	8,7-10,5
Pública	58.707	8.382.253	28,3	12,4	11,4-13,4	8,5	7,9-9,0
Brasil	74.589	10.147.700	27,1	13,1	12,1-13,9	8,7	8,2-9,1

A prevalência geral de asma ativa foi 13,1% (IC95% 12,1-13,9), sendo superior no sexo feminino (14,8%; IC95% 13,7-16,0) em relação ao masculino (11,2%; IC95% 10,3-12,2) e nos alunos da rede privada (15,9%; IC95% 14,2-17,7) em relação àqueles de escolas públicas (12,4%; IC95% 11,4-13.4). Essas taxas foram semelhantes em ambas as faixas etárias estudadas. A prevalência geral de diagnóstico médico de asma foi 8,7% (IC95% 8,2-9,1), sem diferenças significativas entre os sexos, faixa etária e cor da pele. Assim como a asma ativa, a sua prevalência foi mais elevada entre adolescentes da rede privada de ensino, porém sem alcançar significância estatística em relação aos estudantes de escolas públicas (Tabela 1).

A prevalência de asma ativa foi maior na região Sudeste (14,5%; IC95% 12,9-16,1) e nas cidades de São Paulo (16,7%; IC95% 14,7-18,7); Belo Horizonte (15,8%; IC95% 13,9-17,7) e Goiânia (15,4%; IC95% 13,1-17,7). Já a região Norte exibiu a menor prevalência entre as macrorregiões (9,7%; IC95% 9,7-10,5), assim como as cidades de Teresina (6,3%; IC95% 4,9-7,7); São Luís (7,4%; IC95% 6,0-8,8) e João Pessoa (7,8%; IC95% 6,4-9,2). A prevalência de asma ativa foi maior no sexo feminino em todas as capitais e regiões do Brasil (Tabelas 1 e 2).

**Tabela 2:** Prevalência (%) de asma ativa em adolescentes de 12 a 17 anos por sexo, segundo as capitais brasileiras. ERICA, 2013-2014.

Capital	Amostra	População estimada	Asma ativa		Feminino		Masculino	
		
			%	IC95%	%	IC95%	%	IC95%
Porto Velho	1.349	5.2526	11,0	8,1-13,9	12,9	9,5-16,3	9,1	5,3-12,8
Rio Branco	1.740	39.603	8,9	7,4-10,4	12,3	10,1-14,5	5,4	4,0-6,9
Manaus	3.549	214.042	10,2	9,1-11,3	12,4	10,6-14,2	8,0	6,7-9,3
Boa Vista	750	35.813	11,5	8,4-14,7	12,7	8,1-17,2	10,4	5,0-15,8
Belém	2.329	138.765	10,6	9,0-12,2	12,0	10,3-13,8	9,1	7,1-11,1
Macapá	1.370	52.573	12,9	10,7-15,1	16,4	13,0-19,9	9,2	6,6-11,8
Palmas	1.170	27.928	10,9	8,3-13,6	12,6	9,0-16,2	9,3	6,6-11,9
São Luís	2.577	105.548	7,4	6,0-8,8	8,6	7,0-10,1	6,2	4,2-8,2
Teresina	1.733	83.365	6,3	4,9-7,7	8,0	5,8-10,3	4,6	2,9-6,4
Fortaleza	2.665	244.925	10,3	9,0-11,6	12,1	10,3-13,9	8,5	6,5-10,4
Natal	1.944	79.819	10,9	8,5-13,2	13,6	11,2-16,0	8,2	5.4-10,9
João Pessoa	1.956	70.050	7,8	6,4-9,2	9,0	7,2-10,80	6,7	4,9-8,4
Recife	2.534	896.251	9,6	7,3-11,9	9,8	9,3-10,2	3,5	3,1-3,9
Maceió	2.082	101.491	8,0	6,4-9,7	9,7	7,3-12,2	6,4	4,4-8,4
Aracaju	1.788	59.576	9,4	7,2-11,7	10,3	6,8-13,9	8,6	6,3-10,8
Salvador	1.890	252.397	8,4	6,4-10,5	10,2	7,6-12,8	6,6	3,9-9,4
Campo Grande	1.223	78.769	11,0	8,6-13,4	11,6	8,4-14,9	10,3	6,4-14,2
Cuiabá	1.910	52.153	11,5	9,8-13,2	13,6	11,3-15,9	9,6	6,9-12,2
Goiânia	1.598	125.057	15,4	13,1-17,7	16,4	13,9-18,9	14,5	10,5-18,4
Brasília	2.689	268.118	14,8	12,6-17,1	16,2	14,2-18,3	13,4	9,8-17,0
Belo Horizonte	2.569	203.990	15,8	13,9-17,7	19,0	6,7-21,3	12,6	10,5-14,8
Vitória	1.372	29.237	11,7	9,8-13,5	12,4	10,0-14,9	10,9	8,2-13,5
Rio de Janeiro	3.516	516.063	8,9	7,7-10,0	10,7	8,6-12,7	7,1	5,2-9,0
São Paulo	3.700	980.486	16,7	14,7-18,7	19,8	16,3-23,3	13,6	12,0-15,2
Curitiba	2.532	156.140	14,6	13,1-16,2	15,8	13,5-18,0	13,5	10,9-16,1
Florianópolis	1.145	36.420	12,5	9,0-16,0	15,0	10,8-19,2	10,0	6,7-13,2
Porto Alegre	1.948	114.959	14,9	12,9-16,8	17,6	14,4-20,9	12,2	9,9-14,4

Em relação ao diagnóstico médico de asma, a prevalência mais elevada foi observada na região Norte (13,5%; IC95% 12,7-14,2) e nas cidades de Porto Alegre (19,8%; IC95% 17,5-22,3), Belém (15,7%; IC95% 13,5-17,8) e Vitória (15,5%; IC95% 12,6-18,3). Por outro lado, a região Centro-Oeste exibiu a menor prevalência entre as macrorregiões (6,9%; IC95% 6,0-7,8), assim como as cidades de Cuiabá (4,8%; IC95% 3,8-5,9); Campo Grande (5,4%; IC95% 4,2-6,6) e João Pessoa (6,5%; IC95% 5,2-7,7) (Tabela 1 e Figura 1).

**Figura 1:** Prevalência (%) e IC95% de asma diagnosticada pelo médico em adolescentes de 12-17 anos segundo as capitais brasileiras. ERICA, 2013-2014.

A distribuição de prevalência do diagnóstico médico de asma pelo sexo variou amplamente entre os diferentes estratos geográficos estudados. Essas taxas foram mais elevadas no sexo masculino nas regiões Norte (14,6% *versus* 12,9%), Nordeste (9,9% *versus* 8,2%), Centro-Oeste (7,9% *versus* 5,9%) e Sul (10,7% *versus* 10,1%), enquanto no Sudeste houve predomínio entre as meninas (7,8% *versus* 7,3%), sem alcançar significância estatística. Entretanto, na amostra total o diagnóstico médico de asma foi significativamente superior no sexo feminino (14,8%; IC95% 13,7-16,0) em relação ao masculino (11,2%; IC95% 10,3-12,2).

Em relação à cor da pele, a prevalência de asma ativa foi superior naqueles de cor branca (14,9%; IC95% 12,9-16,8) em relação a pretos (10,8%; IC95% 9,2-12,3) e pardos (12,1%; IC95% 11,2-12,9), não ocorrendo diferenças na distribuição da prevalência do diagnóstico médico de asma.

## DISCUSSÃO

Os resultados do ERICA mostraram prevalência média de asma ativa de 13,1% em adolescentes de 12-17 anos, oscilando entre 6,3% em Teresina e 16,7% em Campo Grande, e com predominância entre o sexo feminino em todos os estratos geográficos. Com relação às regiões, houve variação entre 9,7% na região Norte e 14,5% na Sudeste.

A prevalência média de asma diagnosticada por médico nos municípios de mais de 100 mil habitantes no Brasil foi 8,7%, com variação de 6,9% na região Centro-Oeste até 13,5% na região Norte. Essa diferença foi ainda mais acentuada entre as capitais, de 4,8% em Cuiabá a 19,8% em Porto Alegre, porém sem diferença entre os sexos. O percentual de não respostas ao questionário do estudo foi considerado satisfatório e sua variabilidade pode ter sido decorrente da obrigatoriedade do termo de consentimento livre e esclarecido para alguns estados, nos quais foram observados percentuais de participação mais baixos.

A análise comparativa entre os dados aqui obtidos e os previamente observados pelo estudo ISAAC fase III em algumas localidades brasileiras mostrou redução nas taxas de prevalência de asma ativa e de asma diagnosticada por médico^20^. Em estudo realizado posteriormente (em 2012), Solé et al., utilizando o mesmo método, verificaram a prevalência de asma em adolescentes de 13-14 anos de sete cidades brasileiras participantes do ISAAC fase III em 2003 e sua tendência temporal após nove anos do estudo. Nesse período, foi observada queda da prevalência média de asma ativa (19,5% *versus* 17,5%) com elevação da asma diagnosticada pelo médico (14,3% *versus* 17,6%), ambas superiores àquelas obtidas pelo ERICA^23^.

Na PeNSE 2012, foram observadas taxas de 23,2% e 12,4%, respectivamente, para asma ativa e diagnóstico médico de asma. De modo similar aos nossos resultados, os maiores percentuais de asma ativa (24,9% *versus* 14,5%) e de diagnóstico médico de asma (18,4% *versus* 13,5%) foram observados respectivamente entre escolares das regiões Sudeste e Norte. Por outro lado, diferentemente do ERICA, os menores percentuais para estas prevalências na PenSE 2012 ocorreram respectivamente nas regiões Nordeste (19,8%) e Sudeste (11,4%)^2^.

As prevalências de asma ativa e diagnóstico médico de asma no Brasil, em sete capitais brasileiras avaliadas pelo ERICA, e participantes do ISAAC fase III e reavaliadas em 2012, e também incluídas na PeNSE são demonstradas nas Figuras 2 e 3.

Análise superficial possibilitaria concluir sobre a redução temporal dessas taxas após elevação prévia significativa (Figura 2); entretanto, alguns fatores devem ser considerados na análise. Em primeiro lugar, a faixa etária avaliada pelo ERICA é mais ampla que a do ISAAC e da PeNSE, e o ingresso de adolescentes mais velhos poderia gerar diluição nas taxas observadas. Entretanto, não verificamos diferenças significativas entre as taxas de prevalência de asma ativa entre os participantes do ERICA maiores de 15 anos e aqueles entre 12 e 14 anos, mais próximos das populações dos outros estudos citados.

**Figura 2:** Prevalência (%) de asma ativa em adolescentes participantes do ISAAC fase III (2003-2012), PeNSE e ERICA.

ISSAC: *International Study of Asthma and Allergies in Childhood*; PeNSE: Pesquisa Nacional em Saúde do Escolar; ERICA: Estudo de Riscos Cardiovasculares em adolescentes

Outro aspecto refere-se ao modo como foi reconstruída a questão sobre asma ativa em nosso estudo, diferente do apresentado no módulo de asma do ISAAC^1^. A pergunta original sobre a presença de “sibilos nos últimos 12 meses”, útil para avaliação de prevalência de asma^1^ ^,^ diferiu da nossa sobre o número de “crises de sibilos nos últimos 12 meses”. A frequência de crises no último ano, presente como questão independente no módulo de asma do ISAAC, é considerada de grande utilidade para avaliação da gravidade da doença, oferecendo uma alternativa de medida quantitativa da frequência de sibilância^1^. Deste modo, ao incluirmos o termo “crise” para o diagnóstico epidemiológico de asma ativa, a sensibilidade para esta questão pode ter sido reduzida em relação à original, o que pode dificultar a comparação de nossos resultados com os de outros estudos. Por outro lado, aqueles que responderam positivamente a esta pergunta podem representar uma parcela de adolescentes com quadro clínico de maior gravidade ou pior controle da doença, necessitando de maiores cuidados médicos e exercendo maior carga sobre o sistema de saúde.

A dificuldade no diagnóstico de quadros clínicos mais leves de asma em adolescentes pode gerar incerteza sobre a real extensão da doença nesta faixa etária^3^. Estudos longitudinais têm demonstrado que percentual significativo de indivíduos, em geral aqueles com quadro de asma mais grave na infância, permanecem sintomáticos durante a adolescência e serão adultos asmáticos^11^ ^.^ Atualmente, diferentes fenótipos de asma têm sido reconhecidos inclusive de início na adolescência, desafiando o senso comum de que toda a asma tem origem atópica e melhora durante a puberdade^7^. Além disso, quando trabalhamos com adolescentes, devemos atentar para alguns aspectos não especificamente ligados à asma, como o grau de percepção de saúde e doença, bem como as diferenças cognitivas entre os sexos que podem influenciar os resultados de estudos nessa população.

A prevalência de diagnóstico médico de asma obtida pelo ERICA também foi menor quando comparada aos resultados do ISAAC e PeNSE (Figura 3). Entretanto, a relação entre prevalência de asma ativa, mais elevada, e de diagnóstico médico de asma mantém sua proporção nos quatro estudos, o que está de acordo com outros estudos nacionais e internacionais^3^ ^,^ ^15^.

**Figura 3:** Prevalência (%) de diagnóstico médico de asma em adolescentes participantes do ISAAC fase III (2003-2012), PeNSE e ERICA.

ISSAC: *International Study of Asthma and Allergies in Childhood;* PeNSE: Pesquisa Nacional em Saúde do Escolar; ERICA: Estudo de Riscos Cardiovasculares em adolescentes

A resposta positiva à pergunta “Alguma vez na vida você teve asma?”, original do módulo de asma do protocolo ISAAC, é considerada como um possível diagnóstico realizado por médico para essa condição^1^. A diferença entre essa taxa e a de asma ativa tem sido interpretada como um subdiagnóstico médico da asma, o que poderia acarretar na demora ou falta de tratamento da doença^3^. Outro aspecto que pode interferir na interpretação dessa questão é a variação na terminologia e nos conceitos de uma mesma doença para diferentes populações e profissionais de saúde, muitas vezes influenciada por fatores culturais^a^. No ERICA foi perguntado: “Algum médico lhe disse que você tem asma?”, pergunta mais objetiva que pode justificar a sua prevalência mais baixa em comparação com os demais estudos apresentados na Figura 3. O percentual de respostas positivas a esta pergunta em nosso estudo pode traduzir de modo mais preciso a real dimensão ao acesso a serviços médicos e justificar a grande variação encontrada para essa taxa entre as capitais estudadas.

À semelhança de outros estudos^9^ ^,^ ^13^ sem a amplitude do ERICA, observou-se maior prevalência de asma ativa entre adolescentes do sexo feminino. Em geral, meninos apresentam maior chance de desenvolver sibilância e asma nos primeiros anos de vida; porém, esse risco reduz gradualmente até o final da infância, época em que a doença passa a ser mais prevalente entre as meninas. Embora reconhecida, a associação entre o sexo feminino e asma na adolescência não tem até o momento uma causa bem estabelecida. Possíveis fatores como o calibre das vias aéreas, influências hormonais, excesso de peso nos anos pré-escolares, aspectos psicossociais e exposições ambientais próprias de cada sexo têm sido citados. Além disso, estudos epidemiológicos com adolescentes têm demonstrado possíveis diferenças no nível de interesse para preenchimento de questionários, com os meninos tendendo a subestimar e as meninas a superestimar seus sintomas^9^.

Em contraste com os resultados da PeNSE^2^ ^,^ em que não foram observadas diferenças significativas entre estudantes de escolas públicas e privadas, encontramos percentual maior de asma ativa entre os adolescentes de escolas privadas. No ERICA, cuja maioria da amostra é oriunda de escolas públicas, o ingresso em escola privada pode significar melhor condição financeira familiar e maior acesso aos serviços de saúde, ou até mesmo melhor percepção da doença com maior interesse em participar do estudo. Embora não tenha sido encontrada associação significativa entre o nível socioeconômico da amostra e a prevalência da doença em cidades brasileiras participantes do ISAAC III^22^ ^,^ nossos resultados podem refletir as desigualdades sociais nessa população.

Em um País como o Brasil, de dimensões continentais, diferenças regionais, culturais e socioeconômicas podem coexistir com grande variação de fatores ambientais. Estudo realizado em sete cidades da região Nordeste participantes do ISAAC fase III avaliou a associação entre a presença de asma ativa e diferentes indicadores socioeconômicos e condições ambientais. Foi encontrada associação positiva entre a prevalência de asma ativa e privação de água e a latitude do centro, e associação inversa com a média anual de temperatura^8^ ^.^ Do mesmo modo, observamos aumento da prevalência de asma ativa no sentido norte-sul do País.

Os resultados do ERICA mostram que a prevalência de asma permanece muito elevada em adolescentes brasileiros, constituindo importante problema de saúde pública nessa faixa etária. As taxas de prevalência de asma ativa e diagnóstico médico da doença variam amplamente nas diferentes regiões e capitais avaliadas pelo estudo. A exploração futura de fatores regionais associados à asma poderá melhorar o entendimento sobre a sua história natural, seus determinantes sociodemográficos e diferenças no acesso ao cuidado médico. Também podem colaborar para a elaboração de programas preventivos e de políticas de saúde voltadas para a doença em adolescentes.

## Footnotes

a Aires ST. Emprego dos termos asma e bronquite em inquérito epidemiológico sobre doença sibilante entre escolares [dissertação]. Rio de Janeiro (RJ): Faculdade de Medicina da Universidade Federal do Rio de Janeiro; 2005.
